# A Novel Approach for the Early Detection of Medical Resource Demand Surges During Health Care Emergencies: Infodemiology Study of Tweets

**DOI:** 10.2196/46087

**Published:** 2024-01-29

**Authors:** Mahakprit Kaur, Taylor Cargill, Kevin Hui, Minh Vu, Nicola Luigi Bragazzi, Jude Dzevela Kong

**Affiliations:** 1 Department of Biology Faculty of Science York University Toronto, ON Canada; 2 Africa-Canada Artificial Intelligence and Data Innovation Consortium Toronto, ON Canada; 3 Department of Computer Science Lassonde School of Engineering York University Toronto, ON Canada; 4 Dalla Lana School of Public Health University of Toronto Toronto, ON Canada; 5 Laboratory for Industrial and Applied Mathematics York University Toronto, ON Canada

**Keywords:** COVID-19, Twitter, social media, medical supply shortage, pandemic, global health, Granger, convergent cross-mapping, causal analysis, intensive care unit bed, ICU bed

## Abstract

**Background:**

The COVID-19 pandemic has highlighted gaps in the current handling of medical resource demand surges and the need for prioritizing scarce medical resources to mitigate the risk of health care facilities becoming overwhelmed.

**Objective:**

During a health care emergency, such as the COVID-19 pandemic, the public often uses social media to express negative sentiment (eg, urgency, fear, and frustration) as a real-time response to the evolving crisis. The sentiment expressed in COVID-19 posts may provide valuable real-time information about the relative severity of medical resource demand in different regions of a country. In this study, Twitter (subsequently rebranded as X) sentiment analysis was used to investigate whether an increase in negative sentiment COVID-19 tweets corresponded to a greater demand for hospital intensive care unit (ICU) beds in specific regions of the United States, Brazil, and India.

**Methods:**

Tweets were collected from a publicly available data set containing COVID-19 tweets with sentiment labels and geolocation information posted between February 1, 2020, and March 31, 2021. Regional medical resource shortage data were gathered from publicly available data sets reporting a time series of ICU bed demand across each country. Negative sentiment tweets were analyzed using the Granger causality test and convergent cross-mapping (CCM) analysis to assess the utility of the time series of negative sentiment tweets in forecasting ICU bed shortages.

**Results:**

For the United States (30,742,934 negative sentiment tweets), the results of the Granger causality test (for whether negative sentiment COVID-19 tweets forecast ICU bed shortage, assuming a stochastic system) were significant (*P*<.05) for 14 (28%) of the 50 states that passed the augmented Dickey-Fuller test at lag 2, and the results of the CCM analysis (for whether negative sentiment COVID-19 tweets forecast ICU bed shortage, assuming a dynamic system) were significant (*P*<.05) for 46 (92%) of the 50 states. For Brazil (3,004,039 negative sentiment tweets), the results of the Granger causality test were significant (*P*<.05) for 6 (22%) of the 27 federative units, and the results of the CCM analysis were significant (*P*<.05) for 26 (96%) of the 27 federative units. For India (4,199,151 negative sentiment tweets), the results of the Granger causality test were significant (*P*<.05) for 6 (23%) of the 26 included regions (25 states and the national capital region of Delhi), and the results of the CCM analysis were significant (*P*<.05) for 26 (100%) of the 26 included regions.

**Conclusions:**

This study provides a novel approach for identifying the regions of high hospital bed demand during a health care emergency scenario by analyzing Twitter sentiment data. Leveraging analyses that take advantage of natural language processing–driven tweet extraction systems has the potential to be an effective method for the early detection of medical resource demand surges.

## Introduction

### Background

The World Health Organization declared COVID-19 a pandemic on March 11, 2020 [1]. The emergence of this pandemic, caused by SARS-CoV-2, led to an unprecedented disruption in the global health care system that exposed and exacerbated existing vulnerabilities in health infrastructure around the world. In particular, the COVID-19 pandemic has had a profound impact on the global medical supply chain, leading to people struggling desperately to access crucial medical resources in the face of case surges and high resource demand [2].

The unprecedented nature of the pandemic and the limited availability of resources, no matter the country, will inevitably lead to the need for prioritizing scarce medical resources to different extents [3,4]. Wealthy countries, such as the United States, experienced shortages of personal protective equipment (PPE) and ventilators [5,6]. This led to the Centers for Disease Control and Prevention developing guidelines for the optimal sourcing of COVID-19 mitigation equipment such as face masks [7]. The pandemic also resulted in an increased strain on hospital capacity around the world. This was especially true for low- and middle-income countries, where health care systems are likely to already be underresourced and stretched thin, making them particularly vulnerable to becoming overwhelmed [8,9]. Considering the potential for future pandemic scenarios and for the recurrence of existing disease outbreaks as new virus variants emerge, the development of accurate real-time methodologies for detecting and forecasting disease impacts is critical for an effective global health response [10,11]. For hospitals that experience volatile demand surges in the face of a finite medical resource supply, timely solutions are required that can allow for rapid and precise decisions to be made regarding resource allocation.

Given that social media are an emerging source for real-time and easily accessible information, there is potential to leverage data from social media for forecasting real-world outcomes [12-15]. Social media platforms and web search data host a wealth of real-time data that broadly reflect the current state of affairs in a particular region [16]. Although the standards of validation for these new data streams are still being validated because they do not have a track record of use, these unconventional data sources have the potential to aid in short- and long-term surveillance, although the surveillance goals must be clearly defined. Studies have found that leveraging social media to identify shortages has the potential to be a cost-effective solution that can be used in real time [17,18]; for instance, Get Us PPE is a grassroots organization that leveraged Twitter (subsequently rebranded as X) to address medical supply shortages in US health care facilities during the first year of the COVID-19 pandemic [19]. Its success in garnering both public and governmental attention to the PPE shortage crisis has demonstrated that Twitter can be a useful tool for mobilizing efforts to address gaps, identifying regional PPE shortages, and informing decision-making in the health care supply chain. In other countries too, such as India, people used Twitter during the pandemic to amplify demands for medical oxygen and intensive care unit (ICU) beds during periods when health care facilities were overwhelmed by case surges [20].

Studies examining the role of social media to glean information about the characteristics of the pandemic note that data derived from social media and search engine data were used to predict new cases in countries such as South Korea [21], the United States [22-24], China [25-27], and Iran [28]. Twitter data have been analyzed to understand the population-level spread of disease [29-31]. Furthermore, forecasting models have been created to track demand for ICU capacity planning in countries such as Chile [32,33], Brazil [34], Colombia [35], the United States [36], India [37], and China [38]. Previous studies have applied convergent cross-mapping (CCM) analysis to explore possible relationships involving antiepidemic measure–related tweets [39], the dynamics of misleading news on Twitter [40], and the identification of the global drivers of influenza [41]. However, to our knowledge, there are limited studies examining the potential of social media, particularly Twitter, to better understand hospital bed demand.

### Objectives

This study aimed to investigate the potential for social media, a relatively novel data stream, to be leveraged as an early warning and detection system for forecasting medical resource shortages. Specifically, this study sought to determine whether the COVID-19 discourse on Twitter could be linked to real-world ICU bed demand. We applied the Granger causality test and CCM analysis to explore whether a causal relationship exists between the volume of negative sentiment COVID-19 tweets and the proportion of ICU bed occupancy in real time in the United States, Brazil, and India. If social media can be successfully leveraged to develop an effective early warning system for forecasting medical resource demand, health care workers and governments may receive real-time insights into pandemic scenarios to inform urgent resource allocation decisions and gain a head start in preparing for demand surges.

## Methods

### Overview

For our analyses, the volume of negative sentiment COVID-19 tweets was compared with ICU bed demand data for each subregion in the United States, Brazil, and India. These 3 countries were selected for this study because they have high cumulative COVID-19 death tolls [42], and they are among the top 4 nations in terms of Twitter users [43]; in addition, publicly accessible validation data on ICU bed demand are available for each country. Three main restrictions in terms of how many patients can be treated at a hospital during the pandemic are available PPE, available ICU beds, and available health care professionals per shift [44]. The number of available ICU beds was selected as the validation parameter for our model.

### Data Sets

For tweets, we used the publicly available Twitter data set *Two Billion Multilingual COVID-19 Tweets with Sentiment, Entity, Geo, and Gender Labels* (TBCOV), which contains >2 billion COVID-19 multilingual tweets, including geographic location and positive, negative, and neutral sentiment labels [45]. From this data set, negative sentiment tweets were selected to capture the volume of negative Twitter discourse surrounding COVID-19 for the United States, Brazil, and India. From this TBCOV data set, for the period from February 1, 2020, to March 31, 2021, a total of 59,832,393 tweets were extracted for the United States, of which 30,742,934 (51.38%) contained negative sentiment. For Brazil, there were 5,343,723 tweets, of which 3,004,039 (56.22%) contained negative sentiment. For India, there were 9,509,766 tweets, of which 4,199,151 (44.16%) contained negative sentiment.

Real-world hospital bed demand was defined as *inpatient_beds_used_covid_coverage* from the US Health Data *COVID-19 Reported Patient Impact and Hospital Capacity by State Timeseries (RAW)* data set [46] for the United States and as *ICU beds needed* from the Institute for Health Metrics and Evaluation *COVID-19 Projections* data set for Brazil and India [47]. The *COVID-19 Projections* data set contains daily information about each region’s need and capacity for hospital beds overall, including ICU beds. In India, similar to Brazil, the greatest medical supply demand during the pandemic was for oxygen cylinders and ICU beds [48]. Amid the pandemic, the insufficient oxygen-manufacturing capacity and the fragmented nature of the Indian health care system made it extremely difficult for people to obtain the supplies they needed in time [49]. Hashtags and sample tweets posted by the Indian public during the pandemic to secure oxygen cylinders and express the urgent need for ICU beds in specific regions have been documented [50,51].

Data collection and analysis were conducted using Python (Python Software Foundation).

### Granger Causality Test Analysis

A time series of each region was generated using the number of negative sentiment tweets per week. This time series was standardized with mean and SD calculated from historical tweet data. Another time series, the ground truth frequency of ICU bed demand, was generated from our preprocessed medical data. In general, all time-series data were binned in intervals of 1 week.

The Granger causality test was used to determine whether past negative tweet frequency contains information that can help forecast ICU bed demand, in addition to the information contained in the past values of ICU bed demand alone [52]. In theory, this test can be applied to a stationary time series. For a nonstationary time series, first or higher difference can be used instead [53,54]. To see whether the time series could satisfy the requirement for the Granger causality test, the augmented Dickey-Fuller (ADF) test, which determines whether a time series is stationary or nonstationary, was used. In our implementation, the functions *grangercausalitytests* and *adfuller* from the *statsmodels* package for Python were used.

### CCM Analysis

The CCM analysis workflow consisted of embedding, cross-mapping, and convergence analysis as well as validation and performance testing. In embedding, the negative sentiment tweets and ICU bed demand for each region were embedded into higher dimensional spaces to capture their underlying dynamics. In cross-mapping, the embedded time series were compared to identify their relationship. In convergence analysis, the results were assessed using statistical measures to determine whether there is a robust relationship between negative sentiment tweets and ICU bed demand.

Put another way, given 2 time series X and Y, their data point entries can be considered to exist in a vector space with x and y axes, and the points over time form a trajectory in the space. Likewise, one can include the time-delayed values of X as new axes, where the vectors can be *<X(t), X(t−3), X(t−6),...>, <X(t−1), X(t−4), X(t−7),...>, etc*.

If the values of X over time do indeed influence or are linked to the values of Y, then a distance-weighted *k*-nearest neighbor model in the *X, X with delay 1, X with delay 2, etc* vector space applied to the same Y (and delay axes) space can have its output converge to the actual observed values of Y, that is, predict the value of Y. If the convergence between *modeled Y from X with delays* and the actual observed values of Y is close, we can say that the model constructed represents the causality relation between X and Y.

In our implementation, the *causal_ccm* package for Python was used.

### Ethical Considerations

Ethics approval was not required for our study because all data and information are publicly available. In addition, all user-identifiable information was excluded from the study results.

## Results

### Overview

From the TBCOV data set, for the period from February 1, 2020, to March 31, 2021, a total of 30,742,934 tweets containing negative sentiment were extracted for the United States; 3,004,039 tweets containing negative sentiment were extracted for Brazil; and 4,199,151 tweets containing negative sentiment were extracted for India. Our results can be categorized into (1) Granger causality test analysis and (2) CCM analysis.

### Granger Causality Test Analysis

#### United States

**Figure 1:**
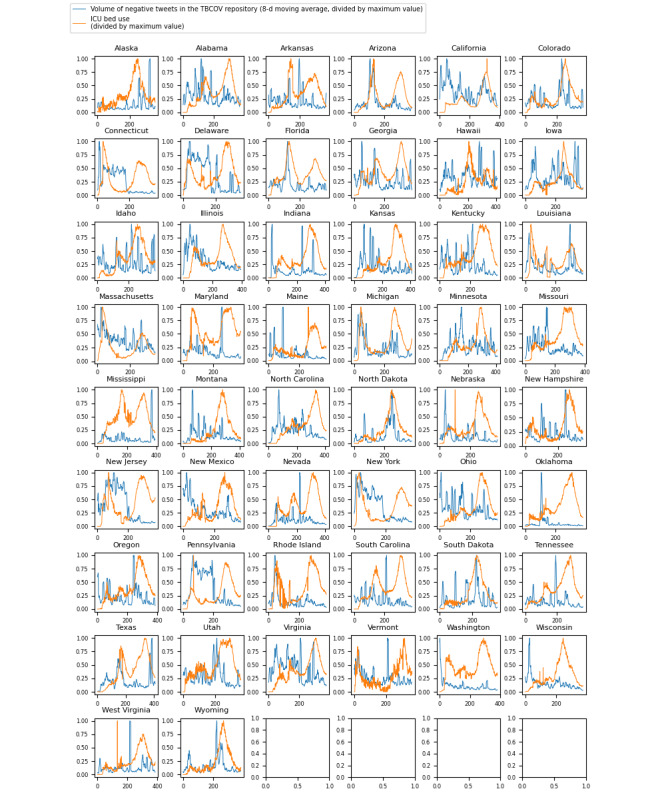
Time series with a comparison of trends for intensive care unit (ICU) bed use with trends for the volume of negative sentiment COVID-19 tweets across all 50 US states. TBCOV: Two Billion Multilingual COVID-19 Tweets with Sentiment, Entity, Geo, and Gender Labels.

Figure 1 shows time series graphs comparing negative sentiment COVID-19 tweets with real-world ICU bed demand data for each of the 50 US states.

Before performing the Granger causality test, the 2 time series were checked to determine whether they were stationary or nonstationary using ADF tests. After taking the second difference of the 2 time series, the *P* values of the ADF tests for negative sentiment COVID-19 tweets and ICU bed demand were found to be <.05 for all US states, meaning that we were able to reject the null hypothesis (H_0_) that a unit root was present in the time series samples; in other words, the 2 time series were stationary. The results are summarized in Multimedia Appendix 1.

The results for the Granger causality test with H_0_, that is, negative sentiment COVID-19 tweets do not Granger-cause ICU bed demand in US states, are presented in Table 1. At lag 2, H_0_ was rejected for 14 (28%) of the 50 US states (*P*<.05).

**Table 1:** Granger causality test for all 50 US states.

State	*P* value	Number of lags	Reject null hypothesis
Alaska	.37	10	No
Alabama	.04	1	Yes
Arkansas	.34	10	No
Arizona	<.001	1	Yes
California	.91	10	No
Colorado	.61	10	No
Connecticut	.99	10	No
Delaware	.40	10	No
Florida	.04	2	Yes
Georgia	.99	10	No
Hawaii	.93	10	No
Iowa	.97	10	No
Idaho	.45	10	No
Illinois	.04	2	Yes
Indiana	.99	10	No
Kansas	.25	10	No
Kentucky	.97	10	No
Louisiana	.10	10	No
Massachusetts	.001	2	Yes
Maryland	.99	10	No
Maine	.95	10	No
Michigan	.02	3	Yes
Minnesota	.59	10	No
Missouri	.43	10	No
Mississippi	.99	10	No
Montana	.32	10	No
North Carolina	.02	4	Yes
North Dakota	.004	3	Yes
Nebraska	.93	10	No
New Hampshire	.01	1	Yes
New Jersey	.93	10	No
New Mexico	.80	10	No
Nevada	<.001	4	Yes
New York	.001	1	Yes
Ohio	.28	10	No
Oklahoma	.047	2	Yes
Oregon	.54	10	No
Pennsylvania	.53	10	No
Rhode Island	.002	1	Yes
South Carolina	.78	10	No
South Dakota	.19	10	No
Tennessee	.94	10	No
Texas	.32	10	No
Utah	.97	10	No
Virginia	.02	7	Yes
Vermont	.70	10	No
Washington	.69	10	No
Wisconsin	.95	10	No
West Virginia	.99	10	No
Wyoming	.24	10	No

#### Brazil

**Figure 2:**
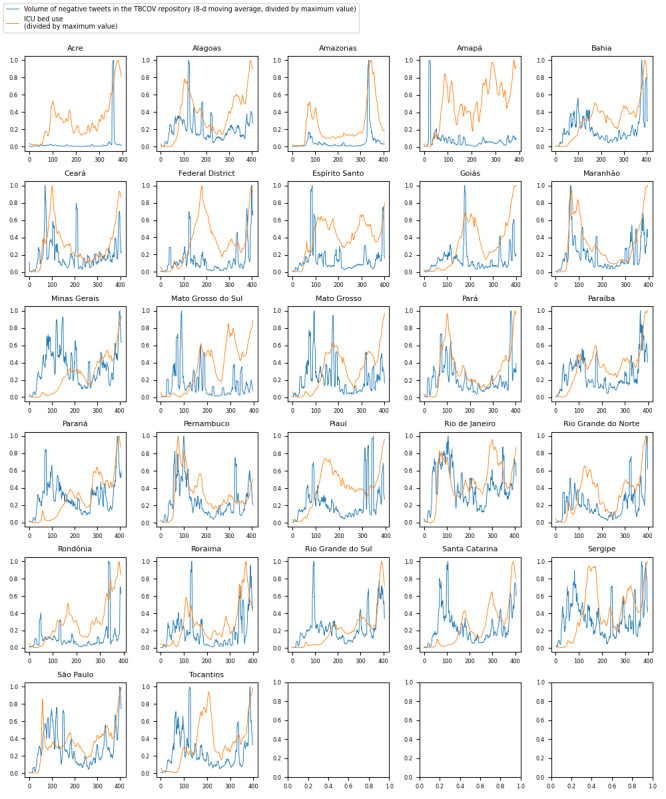
Time series with a comparison of trends for intensive care unit (ICU) bed use with trends for the volume of negative sentiment COVID-19 tweets across Brazil’s 27 subdivisions (states and administrative divisions). TBCOV: Two Billion Multilingual COVID-19 Tweets with Sentiment, Entity, Geo, and Gender Labels.

Figure 2 shows time series graphs comparing negative sentiment COVID-19 tweets with real-world ICU bed demand data for each of the 27 Brazilian federative units.

Before performing the Granger causality test, the 2 time series were checked to determine whether they were stationary or nonstationary using ADF tests, and H_0_ was rejected for all Brazilian federative units (*P*<.05), qualifying all of them for the Granger causality test. The results are summarized in Multimedia Appendix 2.

The results for the Granger causality test with H_0_, that is, negative sentiment COVID-19 tweets do not Granger-cause ICU bed demand in Brazilian federative units, are presented in Table 2. At lag 2, H_0_ was rejected for 6 (22%) of the 27 Brazilian federative units (*P*<.05).

**Table 2:** Granger causality test for Brazilian federative units.

Federative unit	*P* value	Number of lags	Reject null hypothesis
Acre	.99	10	No
Alagoas	.90	10	No
Amazonas	.28	10	No
Amapá	.99	10	No
Bahia	.99	10	No
Ceará	<.001	9	Yes
Federal District	.99	10	No
Espirito Santo	.55	10	No
Goias	.60	10	No
Maranhão	.04	6	Yes
Minas Gerais	.85	10	No
Mato Grosso do Sul	.99	10	No
Mato Grosso	.99	10	No
Pará	.006	2	Yes
Paraíba	.04	1	Yes
Parana	.60	10	No
Pernambuco	.01	2	Yes
Piaui	.90	10	No
Rio de Janeiro	.16	10	No
Rio Grande do Norte	.99	10	No
Rondônia	.91	10	No
Roraima	.002	4	Yes
Rio Grande do Sul	.99	10	No
Santa Catarina	.99	10	No
Sergipe	.48	10	No
São Paulo	.91	10	No
Tocantins	.50	10	No

#### India

**Figure 3:**
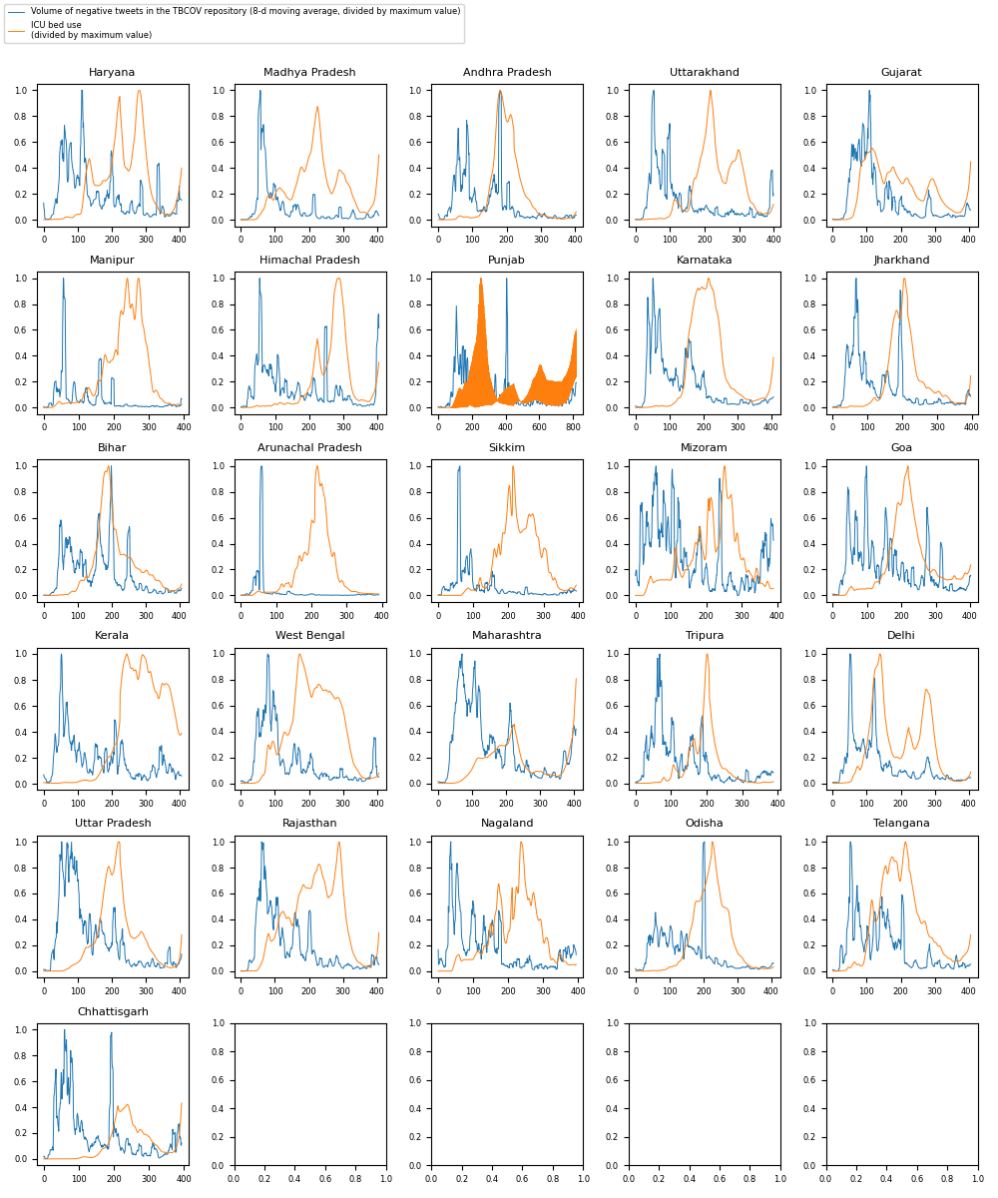
Time series with a comparison of trends for intensive care unit (ICU) bed use with trends for the volume of negative sentiment COVID-19 tweets across Indian states. TBCOV: Two Billion Multilingual COVID-19 Tweets with Sentiment, Entity, Geo, and Gender Labels.

Figure 3 shows time series graphs comparing negative sentiment COVID-19 tweets with real-world ICU bed demand data for the 25 Indian states included in the analysis (Assam, Meghalaya, and Tamil Nadu were excluded owing to lack of data) and the national capital region of Delhi.

Before performing the Granger causality test, the 2 time series were checked to determine whether they were stationary or nonstationary using ADF tests, and H_0_ was rejected for all Indian states and the national capital region of Delhi (*P*<.05), qualifying all for the Granger causality test. The results are summarized in Multimedia Appendix 3.

The results for the Granger causality test with H_0_, that is, negative sentiment tweets do not Granger-cause ICU bed demand in Indian states and the national capital region, are presented in Table 3. At lag 2, H_0_ was rejected for 6 (23%) of the 26 included regions (25 Indian states and the national capital region; *P*<.05).

**Table 3:** Granger causality test for Indian states and the national capital region of Delhi.

State or national capital region	*P* value	Number of lags	Reject null hypothesis
Haryana	.99	10	No
Madhya Pradesh	.60	10	No
Andhra Pradesh	.99	10	No
Uttarakhand	.99	10	No
Gujarat	.86	10	No
Manipur	.003	10	Yes
Himachal Pradesh	.83	10	No
Punjab	.97	10	No
Karnataka	.99	10	No
Jharkhand	.32	10	No
Bihar	.45	10	No
Arunachal Pradesh	.99	10	No
Sikkim	.99	10	No
Mizoram	.99	10	No
Goa	.81	10	No
Kerala	<.001	8	Yes
West Bengal	.28	10	No
Maharashtra	.002	6	Yes
Tripura	.64	10	No
Delhi	.91	10	No
Uttar Pradesh	.99	10	No
Rajasthan	.88	10	No
Nagaland	.99	10	No
Odisha	.98	10	No
Telangana	.99	10	No
Chhattisgarh	.89	10	No

### CCM Analysis

#### United States

**Figure 4:**
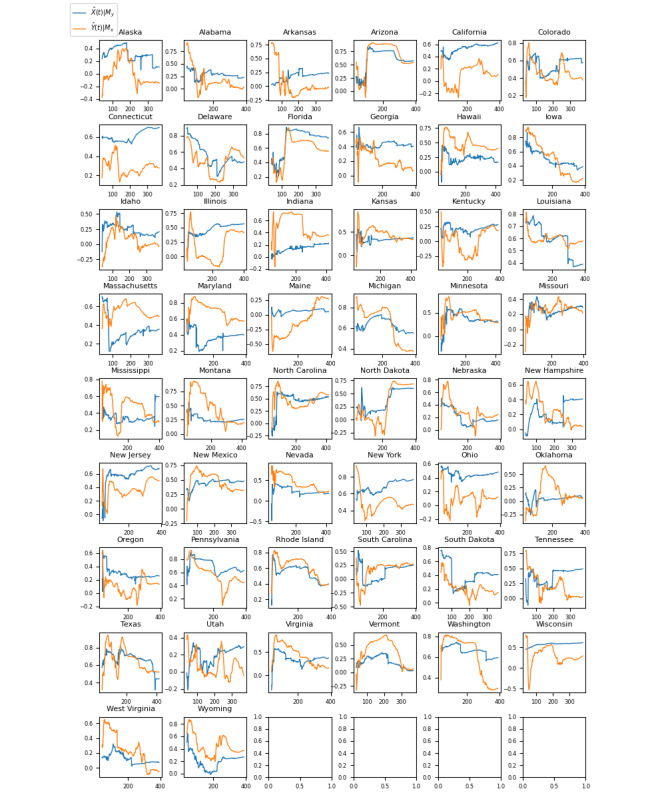
Graphs showing the convergence of the correlation coefficient (r) in convergent cross-mapping analysis as the time series length (L) approaches the maximum possible value for each US state. Series X=negative sentiment COVID-19 tweet proportion and series Y=intensive care unit bed demand. The blue graph represents the correlation coefficient when modeling X causing Y (X→Y). The orange graph represents the correlation coefficient when modeling Y causing X (Y→X). The correlation coefficients ranged from 0.0405 (Vermont) to 0.7670 (New York). The *P* values ranged from .44 (Vermont) to <.001 (New York). Of the 50 US states, 46 (92%) had *P* values <.05.

Figure 4 illustrates the results of the CCM analysis for the United States, where a significant causal relationship was found between ICU bed demand and negative sentiment COVID-19 tweets for 46 (92%) of the 50 states (*P*<.05). The full list of correlation coefficients (*r*) and *P* values for the CCM analysis across US states is presented in Table 4.

**Table 4:** Results of the convergent cross-mapping analysis for each US state. The correlation coefficients (r) ranged from 0.0405 (Vermont) to 0.7670 (New York). Series X=negative sentiment COVID-19 tweet proportion and series Y=intensive care unit bed demand. X→Y refers to the correlation coefficient when modeling X causing Y; Y→X refers to the correlation coefficient when modeling Y causing X.

State	Correlation coefficient, *r*	*P* value	X→Y	Y→X
Alaska	0.1080	.04	0.1080	−0.1520
Alabama	0.2280	<.001	0.2280	0.0211
Arkansas	0.2320	<.001	0.2340	−0.0166
Arizona	0.5740	<.001	0.5730	0.5460
California	0.6270	<.001	0.6260	0.1100
Colorado	0.5750	<.001	0.5760	0.3770
Connecticut	0.7010	<.001	0.7000	0.2770
Delaware	0.4810	<.001	0.4800	0.5290
Florida	0.7430	<.001	0.7430	0.5600
Georgia	0.4050	<.001	0.4040	0.0673
Hawaii	0.1650	<.001	0.1650	0.3960
Iowa	0.3930	<.001	0.3870	0.2240
Idaho	0.2080	<.001	0.2070	−0.0386
Illinois	0.5700	<.001	0.5700	0.4190
Indiana	0.2150	<.001	0.2190	0.3560
Kansas	0.3710	<.001	0.3750	0.3380
Kentucky	0.2790	<.001	0.2790	0.1840
Louisiana	0.3880	<.001	0.3880	0.5810
Massachusetts	0.3590	<.001	0.3570	0.4900
Maryland	0.3980	<.001	0.3980	0.5700
Maine	0.0526	.31	0.0518	0.2660
Michigan	0.5480	<.001	0.5500	0.3720
Minnesota	0.2960	<.001	0.2930	0.2920
Missouri	0.2920	<.001	0.2890	0.2170
Mississippi	0.5950	<.001	0.5950	0.2920
Montana	0.2600	<.001	0.2600	0.2020
North Carolina	0.5470	<.001	0.5400	0.5850
North Dakota	0.6000	<.001	0.5990	0.6830
Nebraska	0.1580	.002	0.1570	0.2520
New Hampshire	0.4080	<.001	0.4080	0.0395
New Jersey	0.6830	<.001	0.6770	0.4970
New Mexico	0.4810	<.001	0.4800	0.3220
Nevada	0.1800	<.001	0.1800	0.2380
New York	0.7670	<.001	0.7660	0.4700
Ohio	0.4840	<.001	0.4830	0.1210
Oklahoma	0.0565	.27	0.0559	0.0657
Oregon	0.2590	<.001	0.2570	0.1350
Pennsylvania	0.6290	<.001	0.6280	0.4440
Rhode Island	0.3990	<.001	0.3990	0.3960
South Carolina	0.2550	<.001	0.2540	0.2780
South Dakota	0.4140	<.001	0.4130	0.1500
Tennessee	0.4920	<.001	0.4920	0.0250
Texas	0.4430	<.001	0.4430	0.5220
Utah	0.2970	<.001	0.2950	−0.0438
Virginia	0.3760	<.001	0.3740	0.1550
Vermont	0.0405	.44	0.0341	0.0602
Washington	0.5930	<.001	0.5930	0.2970
Wisconsin	0.6140	<.001	0.6140	0.2920
West Virginia	0.0746	.14	0.0752	−0.0510
Wyoming	0.2700	<.001	0.2690	0.3740

#### Brazil

**Figure 5:**
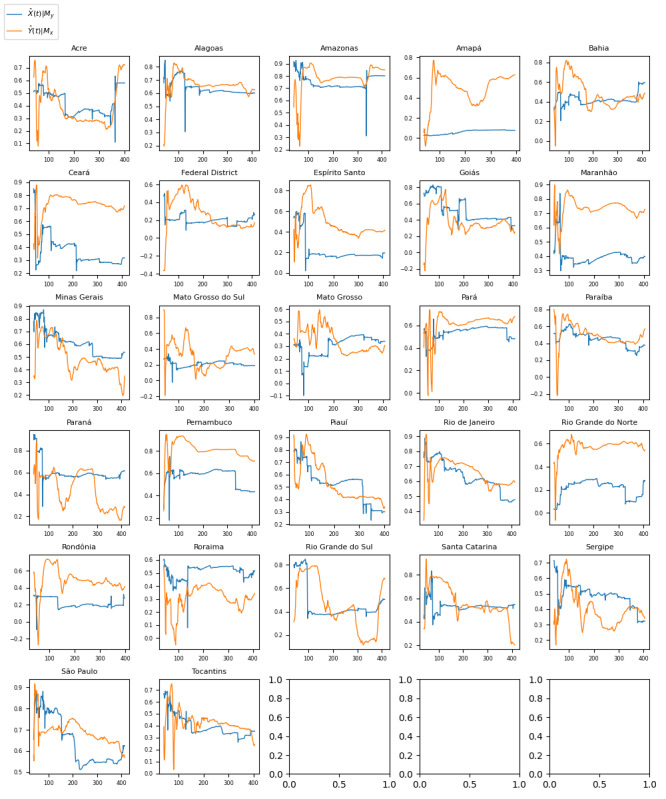
Graphs showing the convergence of the correlation coefficient (r) in convergent cross-mapping analysis as the time series length (L) approaches the maximum possible value for each Brazil subregion. Series X=negative sentiment COVID-19 tweet proportion and series Y= intensive care unit bed demand. The blue graph represents the correlation coefficient when modeling X causing Y (X→Y). The orange graph represents the correlation coefficient when modeling Y causing X (Y→X). The correlation coefficients ranged from 0.0751 (Amapá) to 0.8010 (Amazonas). The *P* values ranged from .14 (Amapá) to <.001 (Amazonas). Of the 27 Brazilian federative units, 26 (96%) had *P* values <.05.

Figure 5 illustrates the results of the CCM analysis for Brazil, where a significant causal relationship was found between ICU bed demand and negative sentiment COVID-19 tweets for 26 (96%) of the 27 Brazilian federative units (*P*<.05). The full list of correlation coefficients (*r*) and *P* values for the CCM analysis across Brazil is presented in Table 5.

**Table 5:** Results of the convergent cross-mapping analysis for each Brazilian federative unit. The correlation coefficients (r) ranged from 0.0751 (Amapá) to 0.8010 (Amazonas). Series X=negative sentiment COVID-19 tweet proportion and series Y=intensive care unit bed demand. X→Y refers to the correlation coefficient when modeling X causing Y; Y→X refers to the correlation coefficient when modeling Y causing X.

Federative unit	Correlation coefficient, *r*	*P* value	X→Y	Y→X
Acre	0.5800	<.001	0.5800	0.7230
Alagoas	0.6010	<.001	0.6010	0.6250
Amazonas	0.8010	<.001	0.8010	0.8500
Amapá	0.0751	.14	0.0758	0.6270
Bahia	0.5940	<.001	0.5940	0.4860
Ceará	0.3140	<.001	0.3170	0.7180
Federal District	0.2720	<.001	0.2600	0.1720
Espirito Santo	0.1940	<.001	0.1940	0.4150
Goias	0.3290	<.001	0.3290	0.2490
Maranhão	0.3970	<.001	0.3960	0.7280
Minas Gerais	0.5370	<.001	0.5360	0.3490
Mato Grosso do Sul	0.1910	<.001	0.1910	0.3350
Mato Grosso	0.3400	<.001	0.3400	0.3070
Pará	0.4750	<.001	0.4830	0.6790
Paraíba	0.3780	<.001	0.3780	0.5710
Parana	0.6110	<.001	0.6150	0.2810
Pernambuco	0.4350	<.001	0.4350	0.7090
Piaui	0.2990	<.001	0.2990	0.3390
Rio de Janeiro	0.4760	<.001	0.4760	0.5930
Rio Grande do Norte	0.2810	<.001	0.2780	0.5440
Rondônia	0.2760	<.001	0.2770	0.4130
Roraima	0.5170	<.001	0.5160	0.3440
Rio Grande do Sul	0.5040	<.001	0.5040	0.6780
Santa Catarina	0.5400	<.001	0.5490	0.2040
Sergipe	0.3230	<.001	0.3220	0.3400
São Paulo	0.6240	<.001	0.6250	0.5690
Tocantins	0.3530	<.001	0.3530	0.2430

#### India

**Figure 6:**
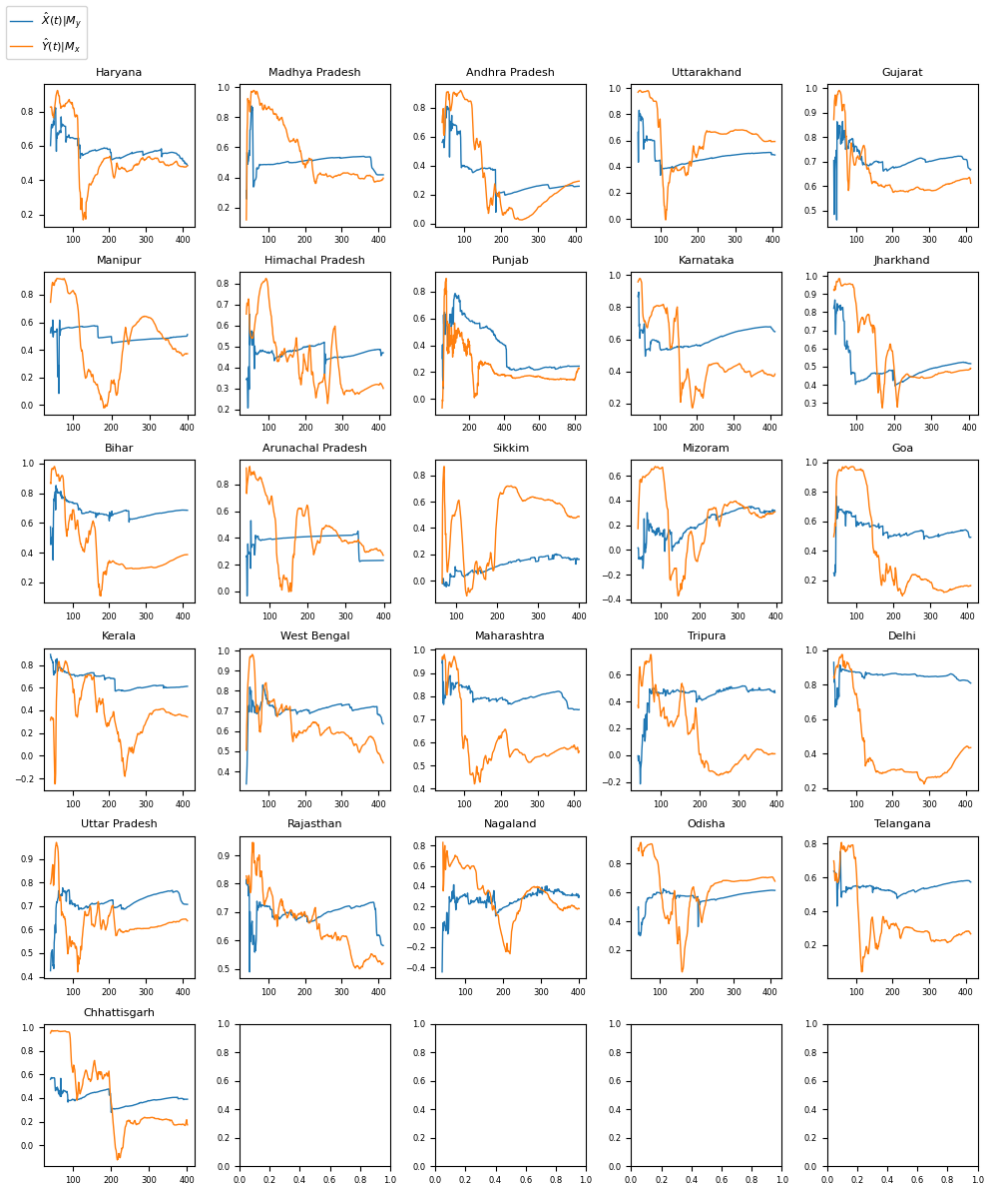
Graphs showing the convergence of the correlation coefficient (r) in convergent cross-mapping analysis as the time series length (L) approaches the maximum possible value for each of the 26 Indian states included in the analysis. Series X=negative sentiment COVID-19 tweet proportion and series Y=intensive care unit bed demand. The blue graph represents the correlation coefficient when modeling X causing Y (X→Y). The orange graph represents the correlation coefficient when modeling Y causing X (Y→X). The correlation coefficients ranged from 0.1630 (Sikkim) to 0.8060 (Delhi). The *P* values ranged from .001 (Sikkim) to <.001 (Delhi). All 25 states and the national capital region of Delhi had *P* values <.05.

Figure 6 illustrates the result of the CCM analysis for India, where a significant causal relationship was found between ICU bed demand and negative sentiment COVID-19 tweets for 26 (100%) of the 26 included regions (25 states and the national capital region; *P*<.05). The full list of correlation coefficients (*r*) and *P* values for the CCM analysis across Indian states and the national capital region is presented in Table 6.

**Table 6:** Results of the convergent cross-mapping analysis for each of the 25 Indian states included in the analysis and the national capital region. Correlation coefficients (r) ranged from 0.163 (Sikkim) to 0.806 (Delhi). Series X=negative sentiment COVID-19 tweet proportion and series Y=intensive care unit bed demand. X→Y refers to the correlation coefficient when modeling X causing Y; Y→X refers to the correlation coefficient when modeling Y causing X.

State or national capital region	Correlation coefficient, *r*	*P* value	X→Y	Y→X
Haryana	0.486	<.001	0.4860	0.4840
Madhya Pradesh	0.417	<.001	0.4170	0.3950
Andhra Pradesh	0.258	<.001	0.2580	0.2940
Uttarakhand	0.489	<.001	0.4890	0.5920
Gujarat	0.669	<.001	0.6680	0.6120
Manipur	0.511	<.001	0.5110	0.3720
Himachal Pradesh	0.470	<.001	0.4700	0.3000
Punjab	0.244	<.001	0.2440	0.2260
Karnataka	0.646	<.001	0.6480	0.3830
Jharkhand	0.517	<.001	0.5170	0.4900
Bihar	0.685	<.001	0.6850	0.3870
Arunachal Pradesh	0.232	<.001	0.2320	0.2700
Sikkim	0.163	<.001	0.1630	0.4870
Mizoram	0.321	<.001	0.3180	0.3080
Goa	0.493	<.001	0.4920	0.1650
Kerala	0.612	<.001	0.6120	0.3420
West Bengal	0.631	<.001	0.6360	0.4420
Maharashtra	0.739	<.001	0.7420	0.5630
Tripura	0.470	<.001	0.4680	0.0105
Delhi	0.806	<.001	0.8090	0.4340
Uttar Pradesh	0.707	<.001	0.7070	0.6370
Rajasthan	0.582	<.001	0.5820	0.5200
Nagaland	0.315	<.001	0.2920	0.1790
Odisha	0.614	<.001	0.6140	0.6770
Telangana	0.573	<.001	0.5730	0.2660
Chhattisgarh	0.390	<.001	0.3900	0.1740

## Discussion

### Principal Findings

Given the need to prioritize the use of limited medical resources during a health care emergency scenario such as the COVID-19 pandemic, social media hold promise in identifying shortages and can be a cost-effective tool for the proper allocation of medical resources, particularly ICU beds, because when governments and organizations are well informed with real-time shortage data, they have the capacity to adequately address the immediate funding and supply needs of health care facilities. This strategy may help mitigate immediate risk to the public until a more systematic solution is possible.

This study sought to determine which patterns existed between negative sentiment COVID-19 tweets and real-world ICU bed shortages during the pandemic, with the aim of leveraging social media to pinpoint regional surges in ICU bed demand in the United States, Brazil, and India. Two statistical tests were conducted to investigate this: the Granger causality test and CCM analysis.

The Granger causality test aims to identify causalities where, in a stochastic system, 1 separable variable is useful for forecasting another. The results of the Granger causality test for this analysis (Figures 1-3 and Tables 1-3) indicate that negative sentiment COVID-19 tweets Granger-caused ICU bed shortage (*P*<.05) for 14 (28%) of the 50 US states, 6 (22%) of the 27 Brazilian federative unit, and 6 (23%) of the 26 Indian regions included in the analysis (25 states and the national capital region). By contrast, the CCM analysis aims to identify causalities for nonseparable variables that are linked in a dynamic system and can identify and quantify weak to moderate causalities that may be missed by the Granger causality test. For the 3 countries, nearly all subregions—46 (92%) of the 50 US states, 26 (96%) of the 27 Brazilian federative units, and 26 (100%) of the 26 Indian regions included in the analysis—had a significant (*P*<.05) result in the CCM analysis (Figures 4-6 and Tables 4-6), indicating a relationship between negative sentiment COVID-19 tweets and ICU bed demand.

For the United States (Tables 1 and 4), of the 50 states, 13 (26%) had a significant result for both the Granger causality test and the CCM analysis (Alabama, Arizona, Florida, Illinois, Massachusetts, Michigan, North Carolina, North Dakota, New Hampshire, Nevada, New York, Rhode Island, and Virginia), 33 (66%) passed the CCM test but not the Granger causality test (Alaska, Arkansas, California, Colorado, Connecticut, Delaware, Georgia, Hawaii, Iowa, Idaho, Indiana, Kansas, Kentucky, Louisiana, Maryland, Minnesota, Missouri, Mississippi, Montana, Nebraska, New Jersey, New Mexico, Ohio, Oregon, Pennsylvania, South Carolina, South Dakota, Tennessee, Texas, Utah, Washington, Wisconsin, Wisconsin, and Wyoming), 1 (2%) passed the Granger causality test only (Oklahoma), and 3 (6%) passed neither the Granger causality test nor the CCM test (Maine, Vermont, and West Virginia). Considering that the majority of the US states (33/50, 66%) passed the CCM test but not the Granger causality test, it can be inferred that the causal relationship between negative sentiment COVID-19 tweets and ICU bed shortage is weak to moderate for US states because CCM analysis is better at detecting weak to moderate causalities than the Granger test. This also implies that the relationship between the 2 variables (social media sentiment and ICU bed shortage) is dynamic and influenced by a number of complex interacting factors such that CCM analysis may be the more appropriate method for detecting and modeling this relationship.

For Brazil (Tables 2 and 5), a similar pattern occurred such that nearly all federative units (26/27, 96%) passed the CCM test, but only a few (6/27, 22%) passed the Granger causality test. Of the 27 federative units, 6 (22%) passed both the Granger causality test and the CCM test (Ceará, Maranhão, Pará, Paraíba, Pernambuco, and Roraima), 20 (74%) passed the CCM test but not the Granger causality test (Acre, Alagoas, Amazonas, Bahia, Federal District, Espirito Santo, Goias, Minas Gerais, Mato Grosso do Sul, Mato Grosso, Parana, Piaui, Rio de Janeiro, Rio Grande do Norte, Rondônia, Rio Grande do Sul, Santa Catarina, Sergipe, São Paulo, and Tocantins), none passed the Granger causality test only, and 1 (4%) state passed neither test (Amapá). This result again supports the idea of a dynamic and complex causal relationship being detected between negative sentiment COVID-19 tweets and ICU bed shortage.

For India (Tables 3 and 6), a similar pattern emerged. Of the 26 regions included in the analysis (25 states and the national capital region), 3 (12%) passed both the Granger causality test and the CCM test (Manipur, Kerala, and Maharashtra), whereas the remaining 23 (88%) passed the CCM test only (Haryana, Madhya Pradesh, Andhra Pradesh, Uttarakhand, Gujarat, Himachal Pradesh, Punjab, Karnataka, Jharkhand, Bihar, Arunachal Pradesh, Sikkim, Mizoram, Goa, West Bengal, Tripura, Delhi, Uttar Pradesh, Rajasthan, Nagaland, Odisha, Telangana, and Chhattisgarh), meaning that all 25 states and the national capital region of Delhi passed at least the CCM test. This further demonstrates that CCM analysis is capable of successfully detecting a causal pattern between negative sentiment pandemic tweets and real-world medical resource shortage and that this can potentially be used to pinpoint specific regions that are expected to face surges in medical resource demand at a given time.

Overall, these results suggest that a significant relationship exists between negative sentiment COVID-19 tweets and real-world ICU bed demand at subnational scales and that this relationship can be effectively detected and modeled using CCM analysis. These findings also indicate that the data contained within social media discourse regarding the COVID-19 pandemic can indeed be leveraged to identify and forecast the real-world impacts of the pandemic in the form of ICU bed demand surges. Further optimization of methods for identifying patterns between X sentiment and real-world medical emergencies can support the development of an early warning system for the robust real-time prediction of health care demand surges. Such a system may give health care workers and government decision makers a critical head start when deciding how to most effectively allocate medical resources in a crisis.

### Future Directions

These results open up the possibility to develop tools that can forecast hospital bed demand in certain regions, although further research is required. This modeling approach can have a significant impact in the context of the health care supply chain. Forecasting ability is a potential factor affecting supply chain performance [27-29]. One study found that tweets related to food insecurity were strongly correlated with real food insufficiencies [16]; the authors noted that there is potential for tweet sentiment analysis to be used as a cost-effective early warning system that can help direct food-related interventions. Similarly, our results suggest that there is potential for negative sentiment COVID-19 posts to relate to actual medical resource shortage in regions where people use public discourse platforms such as Twitter (since rebranded as X). Although the current iteration of the methods described in this paper is only relevant to the time before a peak infection in the region, such methods have the potential to advance preparedness measures for future pandemics as they become more robust [31,32].

Digital data sources can aid in the identification of changes in disease activity, and it is worth exploring whether they show better performance in this regard than traditional COVID-19 metrics such as confirmed cases [33]. In low- and middle-income countries, there is potential for social media to act as a cost-effective early warning system to identify priority regions for medical resource allocation in real time. User behavior data can be extracted, given the unique social network parameters of a region, including the language spoken and the preference for 1 social media platform over another [34]. Similar to how a multilingual data set was used in the analyses in this study, Lopreite et al [55] analyzed a data set of tweets posted in various European languages and found that there were early warning signals of COVID-19 outbreaks before public announcements about an outbreak were made. Social networks that are particular to a region can provide user behavior data that can inform early warning detection systems specific to that region; for example, using Baidu search data, Qin et al [56] were able to predict the number of new COVID-19 cases such as fever, *coronavirus*, and *pneumonia*. Their study, along with similar social media–based early warning detection efforts [37-40], shows potential for the creation of a more effective yet affordable model to forecast new cases.

As noted in the previous subsection, there are complex interacting factors that may explain the results observed. Thus, further investigation is needed to unveil the dynamics underpinning the relationship between X negative sentiment pandemic tweets and medical resource demand; for instance, sociopolitical and economic challenges may have had varying influences on the X discourse by affecting public perceptions of COVID-19 management measures across countries. For Brazil (Figure 2), it can be seen that an increased proportion of negative sentiment preceded the ICU bed demand surge. Concern about preventing the proliferation of COVID-19 was among the major emergent X topics in Brazil, and politics also influenced the X landscape [57]. Brazil has a decentralized health system where the federal government finances the states and municipalities that provide health care services. Different levels of government must be able to coordinate effectively, and a failure to do so can lead to a disjunction in the care provided. A study by Neves et al [58] found that key government stakeholders underestimated the magnitude of the pandemic in the early weeks; for example, the Minas Gerais State Health Department posted messages on social media about not suspending the carnival, which is Brazil’s largest festival. When questionable COVID-19 management decisions are made that increase public health risk, such as decisions not to suspend major public events (eg, Brazil’s annual carnival), it is possible for the X discourse to reflect negative public perceptions of these government decisions before their negative impacts on case count and ICU bed demand. Further research is required to better understand the reliability of social media discourse in reflecting current pandemic management landscapes so that web-based public sentiment can accurately forecast pandemic impacts.

Incongruencies among different levels of government may have also contributed to the results observed. The then President of Brazil, Jair Messias Bolsonaro, issued messages that conflicted with those issued by states and municipalities, such as defending hydroxychloroquine as a COVID-19 treatment and countering mask use and physical distancing [59]. At a state-specific level, the governor of Rio de Janeiro state faced charges regarding irregularities in contracts for building emergency field hospitals, which prevented efforts to relieve strain on hospitals [60]. In addition, the state government of São Paulo was being investigated for ventilator fraud, which led to a shortage of ventilators that prevented citizens from accessing life-saving equipment [61]. Overall, the lack of coordinated effort and strategy for dealing with the pandemic contributed to the inconsistent implementation of preventive measures across the states and resulted in confusion for the public. State-specific challenges may have also contributed to the rise in negative sentiment and increased medical resource demand, which can help explain the results observed.

A similar exploration of potential factors for India can shed light on how they contributed to the nuanced and complex relationship observed between negative sentiment COVID-19 tweets and medical resource demand. India emerged as an interesting case study during the pandemic because of the novel use of X by citizens as a way of sourcing ICU beds and ventilators for their loved ones. As citizens found out that there was a shortage of equipment in the hospitals, they took to X in an effort to source medical supplies that were desperately needed. Adherence to government policy is another factor to consider in the Indian X discourse; for example, both COVID-19 waves were associated with nationwide shutdowns, but as the months wore on, people started to adhere less to measures such as masking and physical distancing. This may have contributed to an increase in cases, overwhelming hospitals [62].

Further exploration into region-specific factors as well as social and political contexts will be important for refining our forecasting models and gaining a better understanding of the complex relationship between X negative sentiment pandemic tweets and medical resource demand.

### Limitations

One process in our methodology involved using tweets that already had sentiment labels and analyzing them. However, we are aware that there may not be data sets containing pandemic-related tweets in future contexts. Therefore, 1 recommendation would be to first extract relevant keyword–related tweets and increase accuracy with the help of supervised natural language processing models. A similar study with the aim of predicting medical resource shortages based on tweets was conducted for the state of New York [63]. The method consisted of using supervised learning to find related tweets, which is a more robust method.

One other limitation is that the ICU bed data sets for Brazil and India that were used to validate the model have decreased reliability because they only provide an estimate of the actual data [18], and they may not have accounted for fluctuations in ICU bed supply. However, because there were no other publicly available data sets, it was reasonable to use these data sets.

The use of X data has some important ethical considerations that lie at the intersection of privacy and data collection; for example, social media data collection can be considered a double-edged sword. On the one hand, it provides very valuable data that can increase the possibility of creating important solutions such as a more cost-effective and faster method of gauging PPE shortages and medical resource demand. On the other hand, current artificial intelligence and data collection practices have raised concerns about privacy and the selling of personal data.

### Conclusions

The COVID-19 pandemic has made it clear that adequate demand-based allocation of medical resources and adequate preparedness for surges in hospital admissions are paramount to reduce cases and deaths at the onset of a pandemic. Between the time that a pandemic hits and the time that a vaccine is developed and distributed, it is vital that the medical supply is carefully managed to ensure that all health care facilities have adequate capacity and proper plans for meeting unprecedented medical resource demand surges. This study analyzed negative sentiment COVID-19 tweets that were compared with real-world ICU bed use data. Our results show promise that leveraging social media, particularly X, has the potential to provide a cost-effective relatively rapid method that can inform resource allocation to facilities that need it most.

Further investigation into the potential of X data in the modeling of medical supply shortages may lead to the development of powerful tools that can inform health care decision-making in pandemic scenarios. X causal analysis in shortage forecasting has the potential to be applied broadly in a global context for identifying medical resource demand and informing health care supply chain decisions.

## Appendix

Multimedia Appendix 1 Augmented Dickey-Fuller Test for US states.

Multimedia Appendix 2 Augmented Dickey-Fuller Test for Brazil subregions.

Multimedia Appendix 3 Augmented Dickey-Fuller Test for India states.

## Abbreviations

ADF: augmented Dickey-Fuller
CCM: convergent cross-mapping
ICU: intensive care unit
PPE: personal protective equipment
TBCOV: Two Billion Multilingual COVID-19 Tweets with Sentiment, Entity, Geo, and Gender Labels

## Data Availability

The data sets generated and analyzed during this study are available in the *Two Billion Multilingual COVID-19 Tweets with Sentiment, Entity, Geo, and Gender Labels* repository [46]; the *Institute for Health Metrics and Evaluation* *COVID-19 Projections* repository [48]; and the *COVID-19 Reported Patient Impact and Hospital Capacity by State Timeseries (RAW)* [47].
