# Supplementary material for: A Novel Approach for the Early Detection of Medical Resource Demand Surges During Health Care Emergencies: Infodemiology Study of Tweets
Source: JMIR Form Res. 2024 Jan 29;8:e46087. doi: 10.2196/46087 (PMC10862249; doi:10.2196/46087)
Supplement: Multimedia Appendix 1 [file formative_v8i1e46087_app1.docx]

| Subdivision Name | ADF Statistic - Beds | p value - Beds | Lag used - Beds | ADF Statistic - Tweets | p value - Tweets | Lag used - Tweets | Reject null hypothesis |
| --- | --- | --- | --- | --- | --- | --- | --- |
| Alaska | -1.04E01 | 2.09E-18 | 12 | -9.26 | 1.39E-15 | 17 | Y |
| Alabama | -1.06E01 | 6.24E-19 | 10 | -1.03E01 | 3.96E-18 | 17 | Y |
| Arkansas | -9.36 | 7.80E-16 | 12 | -9.23 | 1.69E-15 | 17 | Y |
| Arizona | -7.79 | 8.17E-12 | 17 | -9.38 | 7.08E-16 | 16 | Y |
| California | -1.22E01 | 1.38E-22 | 8 | -9.96 | 2.38E-17 | 17 | Y |
| Colorado | -1.03E01 | 3.75E-18 | 12 | -1.03E01 | 2.76E-18 | 15 | Y |
| Connecticut | -9.30 | 1.10E-15 | 12 | -8.90 | 1.16E-14 | 17 | Y |
| Delaware | -9.20 | 2.01E-15 | 13 | -9.99 | 1.98E-17 | 17 | Y |
| Florida | -7.88 | 4.86E-12 | 12 | -9.68 | 1.20E-16 | 14 | Y |
| Georgia | -7.59 | 2.58E-11 | 16 | -9.77 | 7.34E-17 | 17 | Y |
| Hawaii | -1.02E01 | 5.07E-18 | 13 | -1.09E01 | 1.56E-19 | 18 | Y |
| Iowa | -1.31E01 | 1.89E-24 | 7 | -1.09E01 | 9.97E-20 | 16 | Y |
| Idaho | -9.31 | 1.08E-15 | 12 | -9.66 | 1.38E-16 | 16 | Y |
| Illinois | -1.32E01 | 1.12E-24 | 7 | -1.19E01 | 4.88E-22 | 14 | Y |
| Indiana | -9.61 | 1.86E-16 | 14 | -9.81 | 5.87E-17 | 18 | Y |
| Kansas | -1.13E01 | 1.21E-20 | 9 | -9.35 | 8.38E-16 | 18 | Y |
| Kentucky | -9.42 | 5.47E-16 | 15 | -9.25 | 1.50E-15 | 17 | Y |
| Louisiana | -9.05 | 4.87E-15 | 12 | -9.15 | 2.71E-15 | 17 | Y |
| Massachusetts | -9.28 | 1.29E-15 | 12 | -9.82 | 5.46E-17 | 16 | Y |
| Maryland | -1.26E01 | 1.55E-23 | 9 | -1.00E01 | 1.83E-17 | 17 | Y |
| Maine | -8.79 | 2.22E-14 | 16 | -9.92 | 2.99E-17 | 17 | Y |
| Michigan | -1.18E01 | 1.10E-21 | 11 | -9.54 | 2.69E-16 | 17 | Y |
| Minnesota | -1.02E01 | 7.60E-18 | 12 | -9.39 | 6.44E-16 | 17 | Y |
| Missouri | -1.06E01 | 5.13E-19 | 13 | -9.41 | 5.93E-16 | 17 | Y |
| Mississippi | -1.14E01 | 7.05E-21 | 12 | -9.70 | 1.09E-16 | 17 | Y |
| Montana | -9.92 | 3.04E-17 | 13 | -1.02E01 | 5.15E-18 | 18 | Y |
| North Carolina | -8.92 | 1.04E-14 | 13 | -9.90 | 3.35E-17 | 17 | Y |
| North Dakota | -9.87 | 4.04E-17 | 13 | -9.76 | 7.57E-17 | 17 | Y |
| Nebraska | -1.04E01 | 1.57E-18 | 13 | -9.59 | 2.09E-16 | 17 | Y |
| New Hampshire | -9.45 | 4.57E-16 | 12 | -9.42 | 5.61E-16 | 17 | Y |
| New Jersey | -9.25 | 1.55E-15 | 12 | -9.32 | 1.02E-15 | 17 | Y |
| New Mexico | -1.19E01 | 5.27E-22 | 12 | -9.50 | 3.55E-16 | 15 | Y |
| Nevada | -9.40 | 6.30E-16 | 9 | -9.79 | 6.37E-17 | 17 | Y |
| New York | -8.80 | 2.17E-14 | 12 | -1.10E01 | 8.62E-20 | 15 | Y |
| Ohio | -9.74 | 8.69E-17 | 12 | -1.03E01 | 4.11E-18 | 17 | Y |
| Oklahoma | -8.63 | 5.81E-14 | 15 | -9.12 | 3.14E-15 | 16 | Y |
| Oregon | -1.30E01 | 2.63E-24 | 9 | -1.03E01 | 4.34E-18 | 17 | Y |
| Pennsylvania | -8.65 | 5.07E-14 | 17 | -1.05E01 | 1.16E-18 | 16 | Y |
| Rhode Island | -9.27 | 1.37E-15 | 15 | -8.96 | 8.28E-15 | 16 | Y |
| South Carolina | -1.33E01 | 8.56E-25 | 8 | -9.65 | 1.42E-16 | 16 | Y |
| South Dakota | -9.24 | 1.64E-15 | 13 | -1.03E01 | 2.97E-18 | 14 | Y |
| Tennessee | -5.98 | 1.86E-07 | 16 | -9.09 | 3.96E-15 | 17 | Y |
| Texas | -6.34 | 2.76E-08 | 18 | -1.12E01 | 1.82E-20 | 18 | Y |
| Utah | -1.08E01 | 2.34E-19 | 13 | -1.01E+01 | 1.37E-17 | 17 | Y |
| Virginia | -1.04E01 | 1.80E-18 | 12 | -9.89 | 3.56E-17 | 16 | Y |
| Vermont | -1.07E01 | 4.59E-19 | 12 | -1.05E+01 | 1.06E-18 | 16 | Y |
| Washington | -1.23E01 | 6.19E-23 | 8 | -9.22 | 1.82E-15 | 16 | Y |
| Wisconsin | -1.01E01 | 1.02E-17 | 13 | -9.01 | 6.10E-15 | 17 | Y |
| West Virginia | -1.04E01 | 1.98E-18 | 15 | -9.83 | 5.11E-17 | 17 | Y |
| Wyoming | -9.84 | 4.76E-17 | 14 | -1.01E+01 | 9.94E-18 | 15 | Y |
