# Supplementary material for: A Novel Approach for the Early Detection of Medical Resource Demand Surges During Health Care Emergencies: Infodemiology Study of Tweets
Source: JMIR Form Res. 2024 Jan 29;8:e46087. doi: 10.2196/46087 (PMC10862249; doi:10.2196/46087)
Supplement: Multimedia Appendix 2 [file formative_v8i1e46087_app2.docx]

| Subdivision Name | ADF Statistic - Beds | p value - Beds | Lag used - Beds | ADF Statistic - Tweets | p value - Tweets | Lag used - Tweets | Reject null hypothesis |
| --- | --- | --- | --- | --- | --- | --- | --- |
| Acre | -9.12 | 3.26E-15 | 6 | -8.90 | 1.17E-14 | 15 | Y |
| Alagoas | -7.71 | 1.29E-11 | 13 | -8.74 | 2.96E-14 | 15 | Y |
| Amazonas | -5.91 | 2.70E-07 | 12 | -9.60 | 1.97E-16 | 15 | Y |
| Amapa | -8.50 | 1.25E-13 | 7 | -8.97 | 8.01E-15 | 16 | Y |
| Bahia | -1.02E+01 | 7.01E-18 | 7 | -9.85 | 4.41E-17 | 16 | Y |
| Ceara | -7.26 | 1.74E-10 | 12 | -8.37 | 2.63E-13 | 17 | Y |
| Federal District | -7.10 | 4.17E-10 | 13 | -9.50 | 3.43E-16 | 14 | Y |
| Espirito Santo | -7.01 | 7.10E-10 | 12 | -1.02E+01 | 5.72E-18 | 16 | Y |
| Goias | -9.88 | 3.74E-17 | 7 | -8.58 | 7.95E-14 | 15 | Y |
| Maranhao | -1.07E+01 | 3.37E-19 | 4 | -8.81 | 1.98E-14 | 15 | Y |
| Minas Gerais | -1.15E+01 | 4.75E-21 | 6 | -9.49 | 3.69E-16 | 17 | Y |
| Mato Grosso do Sul | -1.01E+01 | 1.19E-17 | 6 | -1.01E+01 | 1.03E-17 | 16 | Y |
| Mato Grosso | -1.00E+01 | 1.84E-17 | 7 | -9.89 | 3.66E-17 | 17 | Y |
| Para | -5.33 | 4.76E-06 | 14 | -8.77 | 2.59E-14 | 16 | Y |
| Paraiba | -5.55 | 1.62E-06 | 14 | -8.97 | 7.74E-15 | 16 | Y |
| Parana | -5.24 | 7.38E-06 | 14 | -8.99 | 7.01E-15 | 15 | Y |
| Pernambuco | -1.01E+01 | 1.09E-17 | 4 | -9.91 | 3.27E-17 | 17 | Y |
| Piaui | -8.65 | 5.30E-14 | 14 | -8.72 | 3.35E-14 | 14 | Y |
| Rio de Janeiro | -6.91 | 1.24E-09 | 12 | -7.92 | 3.76E-12 | 17 | Y |
| Rio Grande do Norte | -7.26 | 1.72E-10 | 12 | -9.34 | 9.13E-16 | 16 | Y |
| Rondonia | -5.77 | 5.47E-07 | 14 | -9.14 | 2.91E-15 | 14 | Y |
| Roraima | -7.98 | 2.66E-12 | 13 | -9.44 | 4.91E-16 | 14 | Y |
| Rio Grande do Sul | -5.76 | 5.71E-07 | 14 | -8.87 | 1.43E-14 | 17 | Y |
| Santa Catarina | -6.49 | 1.24E-08 | 13 | -8.36 | 2.88E-13 | 17 | Y |
| Sergipe | -8.86 | 1.48E-14 | 6 | -9.52 | 3.00E-16 | 15 | Y |
| Sao Paulo | -7.89 | 4.40E-12 | 12 | -9.74 | 8.55E-17 | 16 | Y |
| Tocantins | -6.43 | 1.75E-08 | 13 | -9.03 | 5.50E-15 | 16 | Y |
