# Supplementary material for: A Novel Approach for the Early Detection of Medical Resource Demand Surges During Health Care Emergencies: Infodemiology Study of Tweets
Source: JMIR Form Res. 2024 Jan 29;8:e46087. doi: 10.2196/46087 (PMC10862249; doi:10.2196/46087)
Supplement: Multimedia Appendix 3 [file formative_v8i1e46087_app3.docx]

| Subdivision Name | ADF Statistic - Beds | p value - Beds | Lag used - Beds | ADF Statistic - Tweets | p value - Tweets | Lag used - Tweets | Reject null hypothesis |
| --- | --- | --- | --- | --- | --- | --- | --- |
| Haryana | -6.63 | 5.70E-09 | 5 | -9.46 | 4.51E-16 | 18 | Y |
| Madhya Pradesh | -3.44 | 9.81E-03 | 5 | -1.04E+01 | 2.51E-18 | 16 | Y |
| Andhra Pradesh | -1.46E+01 | 3.96E-27 | 3 | -9.71 | 1.03E-16 | 18 | Y |
| Uttarakhand | -4.63 | 1.13E-04 | 16 | -1.03E+01 | 2.80E-18 | 18 | Y |
| Gujarat | -2.95 | 3.97E-02 | 9 | -1.00E+01 | 1.60E-17 | 18 | Y |
| Manipur | -9.11 | 3.47E-15 | 11 | -9.67 | 1.31E-16 | 17 | Y |
| Himachal Pradesh | -6.11 | 9.54E-08 | 14 | -1.04E+01 | 2.28E-18 | 17 | Y |
| Punjab | -1.25E+01 | 2.81E-23 | 21 | -1.23E+01 | 9.83E-23 | 21 | Y |
| Karnataka | -5.15 | 1.14E-05 | 9 | -1.29E+01 | 3.68E-24 | 17 | Y |
| Jharkhand | -4.53 | 1.71E-04 | 6 | -8.75 | 2.82E-14 | 18 | Y |
| Bihar | -3.15 | 2.30E-02 | 16 | -8.73 | 3.21E-14 | 18 | Y |
| Arunachal Pradesh | -9.65 | 1.41E-16 | 13 | -9.90 | 3.46E-17 | 16 | Y |
| Sikkim | -9.19 | 2.20E-15 | 14 | -1.10E+01 | 7.16E-20 | 15 | Y |
| Mizoram | -1.04E+01 | 1.93E-18 | 6 | -1.07E+01 | 3.69E-19 | 17 | Y |
| Goa | -7.08 | 4.80E-10 | 13 | -1.06E+01 | 5.88E-19 | 16 | Y |
| Kerala | -1.51E+01 | 8.46E-28 | 3 | -1.06E+01 | 7.35E-19 | 18 | Y |
| West Bengal | -9.42 | 5.57E-16 | 5 | -1.02E+01 | 6.09E-18 | 18 | Y |
| Maharashtra | -7.20 | 2.43E-10 | 8 | -1.24E+01 | 4.78E-23 | 13 | Y |
| Tripura | -1.58E+01 | 1.14E-28 | 2 | -1.01E+01 | 1.39E-17 | 17 | Y |
| Delhi | -7.58 | 2.77E-11 | 7 | -9.42 | 5.67E-16 | 17 | Y |
| Uttar Pradesh | -5.97 | 1.93E-07 | 6 | -1.17E+01 | 1.73E-21 | 15 | Y |
| Rajasthan | -7.33 | 1.14E-10 | 5 | -9.13 | 3.03E-15 | 18 | Y |
| Nagaland | -1.09E+01 | 1.28E-19 | 8 | -9.12 | 3.25E-15 | 17 | Y |
| Odisha | -1.54E+01 | 3.40E-28 | 2 | -9.96 | 2.37E-17 | 18 | Y |
| Telangana | -8.19 | 7.82E-13 | 7 | -9.97 | 2.24E-17 | 18 | Y |
| Chhattisgarh | -7.31 | 1.31E-10 | 2 | -9.95 | 2.59E-17 | 17 | Y |
